# Inpatient service utilization amongst infants diagnosed with Respiratory Syncytial Virus infection (RSV) in the United States

**DOI:** 10.1371/journal.pone.0317367

**Published:** 2025-01-13

**Authors:** Jason R. Gantenberg, Kathryn D. Thompson, Robertus van Aalst, David M. Smith, Megan Richards, Christopher B. Nelson, William V. La Via, Sandra S. Chaves, Angela M. Bengtson, David A. Savitz, Andrew R. Zullo

**Affiliations:** 1 Department of Health Services, Policy and Practice, Brown University School of Public Health, Providence, RI, United States of America; 2 Department of Epidemiology, Brown University School of Public Health, Providence, RI, United States of America; 3 Department of Community Health Sciences, Boston University School of Public Health, Boston, MA, United States of America; 4 Global Medical Evidence Generation, Sanofi, Swiftwater, PA, United States of America; 5 Merative, Cambridge, Massachusetts, United States of America; 6 Vaccines Medical Affairs, Sanofi, Swiftwater, Pennsylvania, United States of America; 7 New Products and Innovation, Sanofi, Lyon, France; 8 Department of Epidemiology, Emory University, Atlanta, GA, United States of America; 9 Providence VA Medical Center, Providence, Rhode Island, United States of America; University of Buea, CAMEROON

## Abstract

**Introduction:**

Respiratory syncytial virus (RSV) is the leading cause of hospitalization among US infants. Characterizing service utilization during infant RSV hospitalizations may provide important information for prioritizing resources and interventions.

**Objective:**

The objective of this study was to describe the procedures and services received by infants hospitalized during their first RSV episode in their first RSV season, in addition to what proportion of infants died during this hospitalization.

**Methods:**

In this retrospective observational study, we analyzed three different administrative claims datasets to examine healthcare service utilization during RSV hospitalizations among infants. The study population included infants born between July 2016 and February 2020 who experienced an RSV episode during their first RSV season and had an associated inpatient hospitalization. We stratified infants into three comorbidity groups: healthy term, palivizumab-eligible, and other comorbidities. Outcomes included extracorporeal membrane oxygenation, supplemental oxygen use (in-hospital and post-discharge), mechanical ventilation (invasive and non-invasive), chest imaging, infant mortality, length of inpatient stay, intensive care unit (ICU) admission, and number of days in the ICU.

**Results:**

Chest imaging was the most frequently administered procedure during RSV-associated hospitalizations, with approximately 34–38% of infants receiving it. Around one-quarter of infants were admitted to the ICU during their first RSV hospitalization. Median lengths of stay in the hospital were 3–4 days, extending to 4–6 days in the presence of ICU admission. Palivizumab-eligible infants had higher utilization of healthcare services and spent more time in the hospital or ICU compared to healthy infants or those with other comorbidities.

**Conclusions:**

This study provides insights into the utilization of healthcare services during RSV hospitalizations among infants. Understanding service utilization patterns can aid in improved management and resource allocation for infants in the United States, ultimately contributing to better outcomes and reduced healthcare costs overall. However, likely under-ascertainment of ventilation and oxygen-related services in insurance claims remains an impediment to studying these outcomes.

## Introduction

Respiratory syncytial virus (RSV) is a leading cause of hospitalization among infants, and is associated with acute lower respiratory tract infections (LRTI) such as bronchiolitis and pneumonia [1–3]. RSV infection among US children younger than 1 year is still associated with approximately 100 deaths annually, posing a greater risk to them than influenza [4]. Acute LRTI remains a significant cause of morbidity and mortality in children under the age of five globally, accounting for approximately 10% of inpatient hospital admissions [5,6]. In the United States, the rate of RSV-associated hospitalizations (RSVH) has been estimated at 19.4 per 1,000 infants annually, with approximately 80,000 RSV hospitalizations occurring within the first six months of life in 2020 [7,8].

Certain subgroups of children, including preterm infants, those with congenital heart disease (CHD), and those with chronic lung disease (CLD) of prematurity, are at greatest risk of developing severe RSV and associated complications [9]. Preterm infants have higher RSV hospitalization rates compared to full-term infants [10]. Similarly, children with CLD of prematurity have increased odds of RSVH, while hospitalizations associated with congenital heart disease (CHD) remain considerable [11,12]. However, approximately 75% of hospitalization occur in healthy term infants [13–15]. Newborns and infants less than six weeks old face a higher risk of complications resulting from RSV infection. These complications can manifest in various developmental, cardiac, clinical, and dermatological issues [1]. Bronchiolitis and pneumonia are the most common manifestations of RSV LRTIs, often accompanied by complications such as cough and chest wall hyper-expansion [9,16–19]. Severe cases of LRTI can result in respiratory failure, necessitating admission to the intensive care unit (ICU) and ventilation support [18].

Preventing RSV LRTI in infants is crucial in order to prevent consequent health complications [20]. RSVH and associated chronic conditions are associated with increased healthcare costs, primarily due to higher service utilization [21,22]. The financial burden of RSV hospitalizations is substantial: each hospitalization costs $12,000, on average, and contributes to an annual aggregate cost of $472 million in the United States in 2020, a majority of which is financed by Medicaid [21]. Furthermore, a significant proportion of total RSV-related costs is attributed to hospitalizations, particularly to associated ICU admissions and mechanical ventilation utilization [23]. In addition to the economic impact, RSV episodes also result in a reduction in quality of life, with an estimated loss of 6.17 net quality-adjusted life days per episode in the first 60 days of life [24]. Until 2023, upon the introduction of nirsevimab-alip [25], no universally effective treatments or vaccines for RSV existed. Targeted prevention options such as palivizumab and ribavirin were restricted to specific high-risk populations [7,9,26].

Acute LRTI is known to be a leading cause of child mortality in the US—3% to 14% of cases necessitate ICU admission—and infants younger than 1 year experience the highest rates of severe infection [27] Nonetheless, an incomplete picture of service utilization during infant RSVH prevails. To fill this gap, our primary objectives were to describe inpatient service utilization and death associated with RSV LRTI hospitalizations in three large insurance claims databases. Specifically, we aimed to estimate the proportion of hospitalizations involving ICU admission, supplemental oxygen use (in-hospital and post-discharge), mechanical ventilation (invasive and non-invasive), and extracorporeal membrane oxygenation (ECMO), and in-hospital mortality, as well as the length of inpatient stay, and number of days spent in the ICU.

## Methods

### Data and population

The study focused on infants born between July 1, 2016, and February 29, 2020, utilizing three primary datasets: *Merative MarketScan® Commercial Claims and Encounters (CCAE)*, *MarketScan® Multi-State Medicaid (MDCD)*, and *Optum’s de-identified Clinformatics® Data Mart (CDM)*. These datasets provided information on healthcare encounters, diagnoses, procedures, and outcomes for a large population of infants with different insurance types across the United States. Infants were included in the study if they had an inpatient hospitalization associated with their first RSV diagnosis during their first RSV season, as defined in Gantenberg et al., 2022 [15]. An inpatient stay was considered to be RSV-associated if either the start of the inpatient stay occurred within 0–7 days (inclusive) after a qualifying RSV index diagnosis or the RSV index diagnosis occurred at some point during the inpatient stay (**S1 Fig**). Hospitalized infants in the current analysis represent subsets of the analytic samples in a prior analysis of infants followed during their first RSV season, numbering 521,807 in CCAE, 905,114 in MDCD, and 280,275 in CDM at risk of medically attended RSV [28].

In the rare case that an infant had more than one inpatient stay, we restricted analyses to the first stay. Among inpatient stays included in each analysis, if the RSV index diagnosis occurred more than 3 days after the inpatient admission date, we set the inpatient stay to begin on the date of the first RSV diagnosis, which we refer to as “offsetting” the hospitalization start date. In doing so, we aimed to exclude services that may have occurred prior to a nosocomial RSV infection. In the CDM database, we identified inpatient stays using either the CDM confinement ID or by identifying consecutive dates listing any diagnosis attached to the inpatient place of service. In the MarketScan databases, we identified inpatient stays by analyzing claims related to services rendered in a hospital setting and then used the admission and discharge dates to compute length of stay. We considered RSV diagnoses and procedures to be linked to the inpatient setting when the Place of Service was recorded as 21 and the corresponding Current Procedural Terminology® code was not in 99281–99285. As our study involved only secondary analysis of fully deidentified data, the work does not constitute human subjects research and is not subject to institutional review board review.

### RSV index diagnosis definitions

To account for the inherent limitations of ascertaining RSV diagnoses using payment claims, we employed both specific and sensitive index RSV diagnosis definitions. To enhance clinical validity and reduce potential misclassification, the specific index diagnosis included the International Classification of Diseases, Tenth Revision, Clinical Modification (ICD-10-CM) codes for RSV and acute bronchiolitis due to RSV (B974, J121, J205, and J210). In addition to the specific index diagnosis codes, the sensitive index diagnosis expanded the criteria to include codes for unspecified bronchiolitis (J218 and J219). This broader definition aimed to capture potential cases where the RSV diagnosis was not explicitly specified but could be inferred based on the presence of bronchiolitis [15]; similar approaches have been used in previous studies on RSV diagnosis in administrative data, though we stress that our definitions aim to capture uncertainty associated with misclassification in claims [28–30]. See **S1 Table** for full code lists and conditional logic for when B974 was considered a qualifying RSV diagnosis.

### Comorbidity groups

We classified infants as being in one of three mutually exclusive comorbidity groups: Group A represented healthy term infants (*i*.*e*., those without relevant comorbidities), Group B included infants (approximately) eligible for palivizumab prophylaxis, and Group C was comprised of infants with other comorbidities. We considered infants to be in Group A if they had no indication of having been born younger than 37 weeks (**S2 Table**) and did not have any of the comorbidities listed in **S3 Table** recorded at any time during follow-up. We considered infants to be in Group B if they were < 29 weeks’ gestational age (wGA); 29–31 wGA with CLD; ≥ 29 wGA with hemodynamically significant CHD (HS-CHD); or preterm with unknown wGA and CLD or HS-CHD (possibly both). We considered infants to be in Group C if they were 29–31 wGA with neither CLD nor HS-CHD; 32–36 wGA without HS-CHD; preterm with unknown wGA and with neither CLD nor HS-CHD; or ≥ 37 wGA with a comorbid condition but without HS-CHD. These descriptions follow from our prior work [15].

### Other descriptive variables

To characterize the study population, we calculated the distributions of the following categorical variables. *Infant gestational age* at birth comprised six categories: preterm with unknown gestational age (GA), term with unknown GA, unknown, < 29 weeks, 29–31 weeks, 32–36 weeks, and full term (≥ 37 weeks). We assumed that unknown GA indicated ≥ 37 weeks’ gestation. *Sex* was categorized as male, female, or unknown. *RSV season* indicated the specific season in which the RSV diagnosis occurred. We also included *year of birth* (2016–2020) and calendar *month of birth*. Census division categorized infants’ geographic location at birth based on the U.S. Census Bureau classification (East North Central, East South Central, Middle Atlantic, Mountain, New England, Pacific, South Atlantic, West North Central, West South Central, and Other). *Insurance plan type* was categorized as consumer-driven health plans/high-deductible; exclusive provider organization/preferred provider organization; health maintenance organization; an indemnity, point of service with or without capitation; or other. We also calculated the proportion of infants with *comorbid conditions*, *low birth weight*, *CLD*, *hemodynamically significant CHD*, *and pre-season LRTI*. A full list of ICD-10-CM codes used to identify the presence of comorbid conditions resides in **S3 Table**.

### Outcomes

Our binary healthcare service utilization outcomes included receipt of 1) ICU admission, 2) in-hospital supplemental oxygen use, 2) supplemental oxygen use post discharge, 3) invasive mechanical ventilation, 4) non-invasive mechanical ventilation, and 5) chest imaging. We report counts and proportions (%) for binary outcomes. Our continuous healthcare service utilization outcomes included inpatient length of stay (LOS) and days spent in the ICU, which we described using means, medians, and interquartile ranges. The LOS for each inpatient stay and ICU visit was calculated as the end date minus the start date plus one, such that anyone admitted for some length of time was considered to have at least one day in a given setting. Finally, we assumed that an infant death was related to the RSV hospitalization if the date of death recorded in the claims database fell within the dates of the inpatient stay (inclusive).

We stratified all estimates by the three comorbidity groups and estimated 95% confidence limits for proportions and means using the non-parametric bootstrap percentile method [31]. A complete list of diagnosis and procedure codes for all outcomes can be found in the **S4 Table**. For results referring to the CDM database, cell counts and statistics representing fewer than 5 infants are suppressed in accordance with Optum’s privacy restrictions.

### Stability analysis

In the main analysis, we left-truncated inpatient stays if we found that the infant’s RSV index diagnosis occurred more than three days after hospitalization, reasoning that in such cases, the original hospitalization was not likely due to an RSV infection. To gauge the effects of this decision on our estimates, we conducted a stability analysis in which we reanalyzed the data using the original hospital admission date for the inpatient stay.

## Results

### Characteristics of infants hospitalized with respiratory syncytial virus

Under the specific RSV index diagnosis definition, we identified 5,811 hospitalizations in the CCAE database, 13,668 in the MDCD database, and 2,926 in the CDM database, for a total of 22,405 infant hospitalizations. These numbers correspond to approximately 1.3% of infants who were observed through their first RSV season in our prior study [32] having experienced an RSV-related hospitalization. Utilizing the sensitive RSV index diagnosis definition, we identified 8,807 such hospitalizations in the CCAE database, 22,081 in the MDCD database, and 3,621 in the CDM database, respectively. Across index diagnosis definitions and databases, we observed that a higher proportion of hospitalized infants were male, ranging from 55% to 59%, and born at the end of the calendar year (between September and December). Approximately 62%–71% of infants were categorized as healthy full-term infants (Group A), while 6%–11% were considered eligible for palivizumab treatment (Group B), and 23%–27% were identified as having other underlying health conditions (Group C). Census division and payer type varied by commercial database (CCAE versus CDM). Further details about the sample characteristics can be found in **Table 1**.

**Table 1:** Sample characteristics among infants’ first RSV-associated hospitalization during their first RSV season, stratified by RSV index diagnosis definition.

	**CCAE**		**MDCD**		**CDM**	
RSV Definition	Specific	Sensitive	Specific	Sensitive	Specific	Sensitive
	(N = 5,811)	(N = 8,807)	(N = 13,668)	(N = 22,081)	(N = 2,926)	(N = 3,621)
*Variable*	*N (%)*	*N (%)*	*N (%)*	*N (%)*	*N (%)*	*N (%)*
**Year of birth**						
2016	1,217 (20.9)	1,953 (22.2)	2,937 (21.5)	4,710 (21.3)	527 (18.0)	641 (17.7)
2017	1,399 (24.1)	2,224 (25.3)	3,602 (26.4)	5,991 (27.1)	760 (26.0)	978 (27.0)
2018	1,526 (26.3)	2,275 (25.8)	3,212 (23.5)	5,339 (24.2)	756 (25.8)	953 (26.3)
2019	1,615 (27.8)	2,261 (25.7)	3,702 (27.1)	5,706 (25.8)	843 (28.8)	1,002 (27.7)
2020	54 (0.9)	94 (1.1)	215 (1.6)	335 (1.5)	40 (1.4)	47 (1.3)
**Month of birth**						
January	471 (8.1)	699 (7.9)	976 (7.1)	1,548 (7.0)	256 (8.7)	319 (8.8)
February	212 (3.)	365 (4.1)	434 (3.2)	794 (3.6)	89 (3.0)	131 (3.6)
March	92 (1.6)	215 (2.4)	151 (1.1)	365 (1.7)	35 (1.2)	52 (1.4)
April	171 (2.9)	340 (3.9)	191 (1.4)	455 (2.1)	22 (0.8)	38 (1.0)
May	384 (6.6)	613 (7.0)	540 (4.0)	1,100 (5.0)	143 (4.9)	154 (4.3)
June	426 (7.3)	705 (8.0)	707 (5.2)	1,316 (6.0)	130 (4.4)	171 (4.7)
July	463 (8.0)	766 (8.7)	1,196 (8.8)	2,180 (9.9)	236 (8.1)	284 (7.8)
August	566 (9.7)	876 (9.9)	1,502 (11.0)	2,521 (11.4)	281 (9.6)	351 (9.7)
September	674 (11.6)	994 (11.3)	1,820 (13.3)	2,887 (13.1)	350 (12.0)	441 (12.2)
October	846 (14.6)	1,201 (13.6)	2,275 (16.6)	3,365 (15.2)	488 (16.7)	596 (16.5)
November	862 (14.8)	1,147 (13.0)	2,255 (16.5)	3,229 (14.6)	514 (17.6)	614 (17.0)
December	644 (11.1)	886 (10.1)	1,621 (11.9)	2,321 (10.5)	382 (13.1)	470 (13.0)
**Census division**						
East North Central	1,040 (17.9)	1,622 (18.4)	-	-	414 (14.1)	532 (14.7)
East South Central	328 (5.6)	507 (5.8)	-	-	82 (2.8)	113 (3.1)
Middle Atlantic	892 (15.4)	1,275 (14.5)	-	-	212 (7.2)	252 (7.0)
Mountain	491 (8.4)	757 (8.6)	-	-	384 (13.1)	509 (14.1)
New England	210 (3.6)	346 (3.9)	-	-	87 (3.0)	110 (3.0)
Pacific	298 (5.1)	450 (5.1)	-	-	198 (6.8)	247 (6.8)
South Atlantic	1,100 (18.9)	1,689 (19.2)	-	-	490 (16.7)	576 (15.9)
Other	35 (0.6)	55 (0.6)	-	-	16 (0.5)	20 (0.6)
West North Central	578 (9.9)	870 (9.9)	-	-	494 (16.9)	617 (17.0)
West South Central	839 (14.4)	1,236 (14.0)	-	-	549 (18.8)	645 (17.8)
**First RSV season**						
2016–2017	1,465 (25.2)	2,367 (26.9)	3,422 (25.0)	5,586 (25.3)	655 (22.4)	813 (22.5)
2017–2018	1,396 (24.0)	2,189 (24.9)	3,533 (25.8)	5,803 (26.3)	744 (25.4)	951 (26.3)
2018–2019	1,501 (25.8)	2,281 (25.9)	3,165 (23.2)	5,314 (24.1)	747 (25.5)	954 (26.3)
2019–2020	1,449 (24.9)	1,970 (22.4)	3,548 (26.0)	5,378 (24.4)	780 (26.7)	903 (24.9)
**Sex**						
Female	2,521 (43.4)	3,623 (41.1)	6,142 (44.9)	9,500 (43.0)	1,273 (43.5)	1,576 (43.5)
Male	3,290 (56.6)	5,184 (58.9)	7,522 (55.0)	12,575 (56.9)	1,653 (56.5)	2,045 (56.5)
Unknown	-	-	4 (0.0)	6 (0.0)	-	-
**Insurance plan type**						
CDHP/HDHP	1,223 (21.0)	1,881 (21.4)	4,783 (35.0)	7,681 (34.8)	871 (29.8)	1,100 (30.4)
EPO/PPO	3,117 (53.6)	4,642 (52.7)	10 (0.1)	13 (0.1)	315 (10.8)	377 (10.4)
HMO	605 (10.4)	951 (10.8)	0 (0.0)	0 (0.0)	284 (9.7)	350 (9.7)
IND	94 (1.6)	154 (1.7)	8,834 (64.6)	14,315 (64.8)	†	†
OTH	131 (2.3)	194 (2.2)	0 (0.0)	0 (0.0)	38 (1.3)	42 (1.2)
POS	641 (11.0)	985 (11.2)	41 (0.3)	72 (0.3)	1,418 (48.5)	1,752 (48.4)
**Gestational age**						
Preterm, less than 29 weeks	85 (1.5)	275 (3.1)	365 (2.7)	1,046 (4.7)	‡	6 (0.2)
Preterm, 29–31 weeks	132 (2.3)	221 (2.5)	483 (3.5)	825 (3.7)	21 (0.7)	20 (0.6)
Preterm, 32–36 weeks	985 (17.0)	1,471 (16.7)	2,560 (18.7)	4,042 (18.3)	325 (11.1)	409 (11.3)
Preterm, GA Unknown	3,129 (53.8)	4,544 (51.6)	6,805 (49.8)	10,510 (47.6)	231 (7.9)	316 (8.7)
Term, GA Unknown	176 (3.0)	345 (3.9)	390 (2.9)	719 (3.3)	1,666 (56.9)	2,027 (56.0)
Unknown (assume 37+ weeks)	1,304 (22.4)	1,951 (22.2)	3,065 (22.4)	4,939 (22.4)	683 (23.3)	843 (23.3)
**Comorbidity group**						
A. Healthy term	3,985 (68.6)	5,599 (63.6)	9,051 (66.2)	13,609 (61.6)	2,089 (71.4)	2,544 (70.3)
B. Palivizumab-eligible	349 (6.0)	875 (9.9)	977 (7.1)	2,491 (11.3)	173 (5.9)	249 (6.9)
C. Other comorbidities	1,477 (25.4)	2,333 (26.5)	3,640 (26.6)	5,981 (27.1)	664 (22.7)	828 (22.9)
**Low birthweight**						
Low birthweight	744 (12.8)	1,277 (14.5)	2,136 (15.6)	3,934 (17.8)	326 (11.1)	431 (11.9)
**Chronic lung disease**						
Chronic lung disease	85 (1.5)	302 (3.4)	300 (2.2)	999 (4.5)	38 (1.3)	61 (1.7)
**HS-CHD**						
HS-CHD	264 (4.5)	619 (7.0)	614 (4.5)	1,542 (7.0)	140 (4.8)	204 (5.6)
**Any comorbidities**						
Any comorbidities	833 (14.3)	1,774 (20.1)	1,834 (13.4)	4,350 (19.7)	427 (14.6)	572 (15.8)
**MA RSV LRTI in preseason**						
MA RSV LRTI in preseason	10 (0.2)	259 (2.9)	63 (0.5)	764 (3.5)	10 (0.3)	47 (1.3)

Abbreviations: RSV, respiratory syncytial virus; HS-CHD, hemodynamically significant congenital heart disease; COMP, comprehensive; IND, indemnity; EPO, exclusive provider organization; PPO, preferred provider organization; CDHP, consumer-driven health plan; HDHP, high-deductible health plan; POS(WC), point of service (with or without capitation); HMO, health maintenance organization; MA, medically attended; LRTI, lower respiratory tract infection.

† Suppressed due to small cell count and merged into "Other".

‡ Suppressed due to small cell count and merged into "Preterm, 29–31 weeks".

### Outcomes, procedures, and services

Around one-quarter of infants (20.7%–27.2%) were admitted to the ICU, with chest imaging (33.9%–38.1%) and supplemental oxygen (14.7%–15.7%) being the most commonly administered procedures, as detailed in **Table 2**. The occurrence of ECMO usage or in-hospital mortality was less than one percent. Invasive and non-invasive mechanical ventilation were infrequent, with the highest estimated risk across claims databases and RSV index diagnosis definitions being 3.7% (see Discussion for important context regarding this finding). Following hospital discharge, between 1.5% and 4.9% of infants received supplemental oxygen. The healthcare services provided to infants hospitalized during their first RSV episode within their initial RSV season were generally consistent across databases and did not show significant variation based on the RSV index diagnosis definition.

**Table 2:** Proportion of RSV-associated hospitalizations involving a given service/procedure or death, by MA RSV LRTI index diagnosis definition.

Outcome	CCAE			MDCD			CDM^a^		
	N	%	95% CLs^b^	N	%	95% CLs^b^	N	%	95% CLs^b^
**Specific** ^**c**^	**5,811**			**13,668**			**2,926**		
Chest imaging	2,213	38.1	36.8, 39.3	4,729	34.6	33.8, 35.4	1,092	37.3	35.6, 39.1
Died in-hospital	6	0.1	0.0, 0.2	13	0.1	0.0, 0.1	< 5	< 0.1	-
ECMO	5	0.1	0.0, 0.2	17	0.1	0.1, 0.2	< 5	< 0.1	-
ICU admission	1,583	27.2	26.1, 28.4	3,485	25.5	24.8, 26.2	786	26.9	25.3, 28.5
Mechanical ventilation, invasive^d^	38	0.7	0.4, 0.9	111	0.8	0.7, 1.0	108	3.7	3.0, 4.4
Mechanical ventilation, non-invasive^d^	52	0.9	0.7, 1.2	127	0.9	0.8, 1.1	51	1.7	1.3, 2.3
Supplemental oxygen, in-hospital	883	15.2	14.3, 16.1	2,120	15.5	14.9, 16.1	459	15.7	14.4, 17.0
Supplemental oxygen, post-discharge	157	2.7	2.3, 3.1	210	1.5	1.3, 1.7	119	4.1	3.4, 4.8
**Sensitive** ^**c**^	**8,807**			**22,081**			**3,621**		
Chest imaging	3,235	36.7	35.7, 37.7	7,485	33.9	33.3, 34.5	1,301	35.9	34.4, 37.5
Died in-hospital	15	0.2	0.1, 0.3	47	0.2	0.2, 0.3	< 5	< 0.1	-
ECMO	13	0.1	0.1, 0.2	25	0.1	0.1, 0.2	5	0.1	0.0, 0.3
ICU admission	2,066	23.5	22.6, 24.3	4,560	20.7	20.1, 21.2	879	24.3	22.9, 25.7
Mechanical ventilation, invasive^d^	49	0.6	0.4, 0.7	209	0.9	0.8, 1.1	121	3.3	2.8, 3.9
Mechanical ventilation, non-invasive^d^	67	0.8	0.6, 0.9	233	1.1	0.9, 1.2	57	1.6	1.2, 2.0
Supplemental oxygen, in-hospital	1,296	14.7	14.0, 15.5	3,244	14.7	14.2, 15.2	546	15.1	13.9, 16.3
Supplemental oxygen, post-discharge	324	3.7	3.3, 4.1	493	2.2	2.0, 2.4	178	4.9	4.2, 5.6

^a^ Some counts and percentages suppressed due to small cell size. Confidence limits are not shown for these values.

^b^ Non-parametric bootstrap percentile intervals.

^c^ RSV index diagnosis definition.

^d^ See Discussion for major limitations in ascertaining ventilation outcomes in payment claims data.

*Abbreviations*: MA, medically attended; RSV, respiratory syncytial virus; LRTI, lower respiratory tract infectino; CCAE; Merative MarketScan Commercial Claim and Encounters Database; MDCD; Multi-State MarketScan Medicaid; CDM; Optum’s de-identified Clinformatics*®* Data Mart Database; ECMO, extracorporeal membrane oxygenation.

### Length of inpatient stay

Infants had hospital stays with median durations of 3–4 days regardless of insurance claims database or RSV index diagnosis definition (**Table 3**). Interquartile range widths varied from 2 to 5 days for CCAE and MDCD, and from 3 to 6 days for CDM. When infants had an ICU admission, their median inpatient stay was extended by 2–3 days compared to hospitalizations without ICU admission. Among hospitalizations that included an ICU stay, infants spent a median of 4–6 days receiving intensive care, as detailed in **Table 3**.

**Table 3:** Distributions of length of inpatient stay and number of days spent in the intensive care unit (ICU), by MA RSV LRTI index diagnosis definition.

Outcome	CCAE			MDCD			CDM		
	Median	IQR	Mean (95% CLs)^a^	Median	IQR	Mean (95% CLs)^a^	Median	IQR	Mean (95% CLs)^a^
**Sensitive** ^**b**^									
ICU days^c^	5	(3, 8)	6.64 (6.34, 6.97)	5	(4, 8)	7.52 (7.21, 7.85)	4	(3, 6)	5.82 (5.30, 6.45)
Inpatient stay (ICU)	5	(4, 8)	7.53 (7.13, 7.99)	5	(4, 8)	7.74 (7.42, 8.08)	6	(5, 9)	8.83 (8.13, 9.62)
Inpatient stay (no ICU)	3	(2, 4)	4.56 (4.34, 4.79)	3	(1, 4)	4.56 (4.40, 4.74)	3	(3, 5)	4.09 (3.98, 4.20)
Inpatient stay (overall)	3	(2, 5)	5.25 (5.06, 5.46)	3	(2, 5)	5.22 (5.07, 5.38)	4	(3, 6)	5.24 (5.04, 5.46)
**Specific** ^**b**^									
ICU days^c^	5	(3, 8)	6.54 (6.25, 6.84)	6	(4, 8)	7.30 (7.04, 7.58)	4	(3, 6)	5.69 (5.18, 6.36)
Inpatient stay (ICU)	6	(4, 9)	7.24 (6.94, 7.54)	6	(4, 8)	7.43 (7.17, 7.72)	6	(5, 9)	8.60 (7.91, 9.45)
Inpatient stay (no ICU)	3	(2, 4)	3.74 (3.57, 3.96)	3	(1, 4)	3.71 (3.61, 3.82)	4	(3, 5)	4.23 (4.11, 4.37)
Inpatient stay (overall)	3	(2, 5)	4.69 (4.54, 4.87)	3	(2, 5)	4.66 (4.56, 4.77)	4	(3, 6)	5.41 (5.18, 5.66)

^a^ Confidence intervals estimated using the non-parametric bootstrap percentile method (R = 9,999 replicates).

^b^ RSV index diagnosis definition.

^c^ Number of days spent in ICU calculated among inpatient stays featuring at least one visit to the ICU.

Abbreviations: MA, medically attended; RSV, respiratory syncytial virus; LRTI, lower respiratory tract infection; ICU, intensive care unit; CCAE; Merative MarketScan Commercial Claim and Encounters Database; MDCD; Multi-State MarketScan Medicaid; CDM; Optum’s de-identified Clinformatics*®* Data Mart Database.

### Variation by comorbidity group

**Fig 1:**
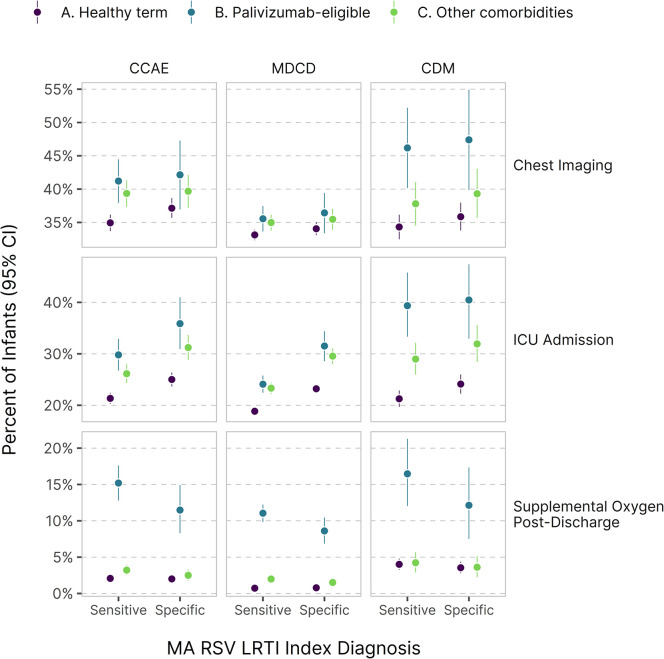
Select primary binary outcomes by comorbidity group. Note that y-axis differs for each panel, as informed by the data range. Color scale refers to infant comorbidity groups. Mechanical ventilation and in-hospital supplemental oxygen use are omitted due to likely data quality issues (see Discussion), while in-hospital death and extracorporeal membrane oxygenation are omitted due to the potential to derive small cell sizes in the Clinformatics data. CCAE, Merative MarketScan Commercial Claim and Encounters Database; MDCD, Multi-State Marketscan Medicaid; CDM, Optum’s de-identified Clinformatics® Data Mart Database.

**Fig 2:**
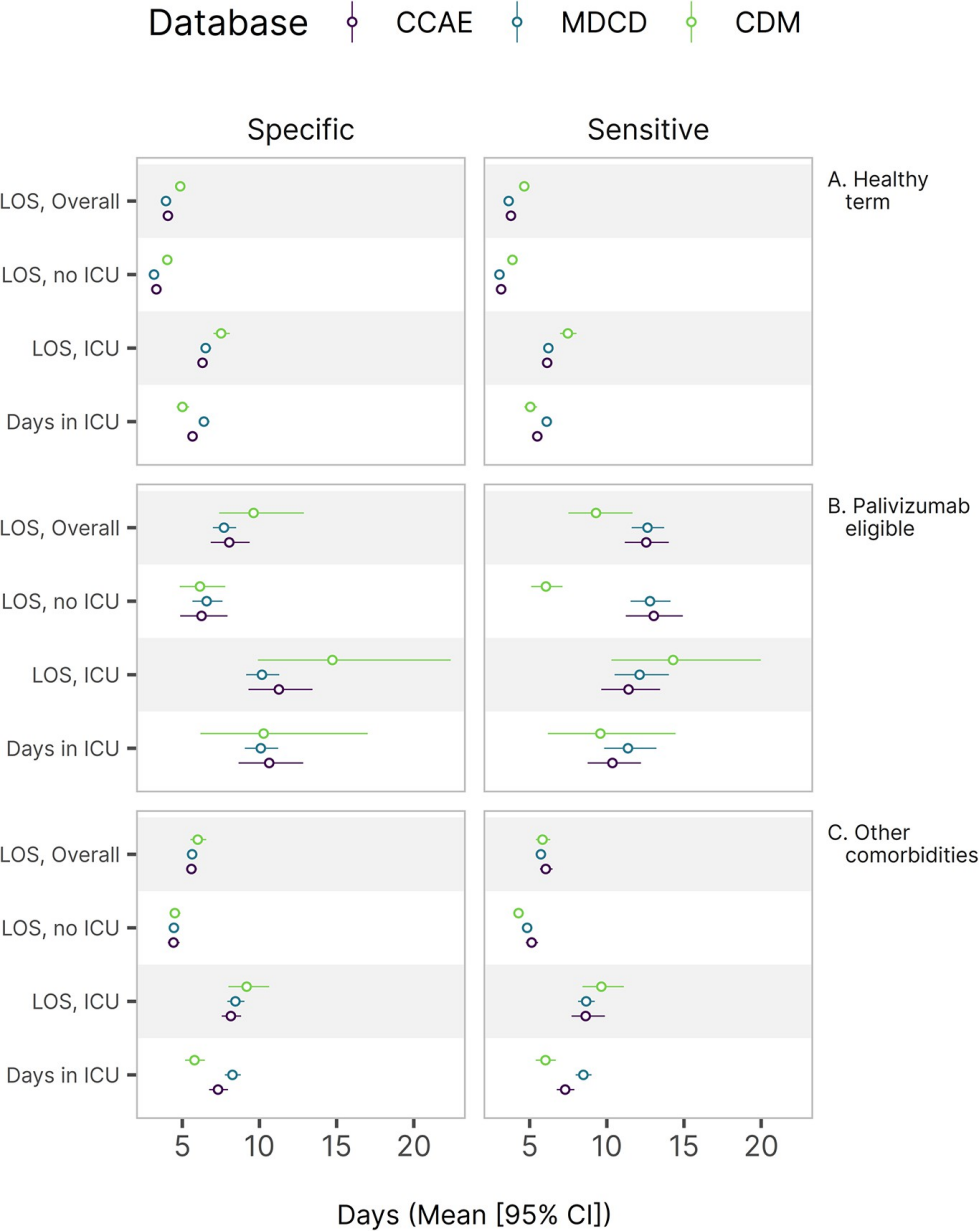
Primary continuous outcomes by comorbidity group. 95% confidence intervals calculated using the non-parametric bootstrap percentile method (R = 9,999 replicates). Colors refer to infant comorbidity groups. CCAE, MarketScan Commercial Claims and Encounters; MDCD, MarketScan Multi-State Medicaid; CDM, Optum’s de-identified Clinformatics® Data Mart Database; RSV, respiratory syncytial virus; LRTI, lower respiratory tract infection; LOS, length of inpatient stay; ICU, intensive care unit.

Palivizumab-eligible infants in comorbidity group B exhibited the highest risks of chest imaging, ICU admission, and post-discharge supplemental oxygen (**Fig 1**). Infants with other comorbidities (group C) also showed increased chest imaging and ICU utilization. All infants had similar in-hospital supplemental oxygen use (numerical values are provided in the supplemental code repository but suppressed in Fig 1 due to suspected underascertainment in claims; see Discussion). The proportion of infants’ utilization healthcare services varied by comorbidity group, with palivizumab-eligible infants and infants with other comorbidities utilizing services more frequently (**Fig 1**). Palivizumab-eligible infants had the longest average length of stay, especially when ICU admission was involved, and they spent more time in the ICU (**Fig 2**).

### Stability analysis

When we relaxed the RSV index date offset—that is, when we used the hospitalization admission date regardless of the timing of the RSV index diagnosis—the marginal results for binary outcomes were almost identical in the commercial databases. In the MDCD, however, the proportion of infants receiving chest imaging and being admitted to the ICU increased by 6–7 percentage points (**S2 Fig**). We observed 5 to 7 percentage-point increases in these same proportions among palivizumab-eligible infants in the CCAE database under the sensitive RSV index diagnosis definition, as well as 4 to 14 percentage-point increases across comorbidity groups in the MDCD database, regardless of the RSV index diagnosis definition, with the largest changes again observed among palivizumab-eligible infants (**S3 Fig**).

Similarly, the influence of alternative decisions for continuous outcome distributions was minor-to-negligible in the commercial databases but increased mean, median, and 75% quantile estimates of the length of inpatient stay and days spent in the ICU within the MDCD database (**S4–S7 Figs**). Our findings were similar across comorbidity groups, though estimates of the mean and 75% quantile were more sensitive among palivizumab-eligible infants within the CCAE and MDCD databases, primarily under the sensitive RSV index diagnosis definition (**S8–S11 Figs**).

## Discussion

In this study, we sought to understand the patterns of service utilization during RSVH in infants, utilizing three large insurance claims databases representing both privately and publicly insured infants in the U.S. Our findings indicate consistent RSV-related inpatient service utilization patterns across all three insurance claim databases we analyzed. Notably, a higher proportion of hospitalized infants were male, and a significant number were born between September and December. These findings corroborate existing knowledge, underlining RSV’s status as a seasonal respiratory pathogen with a predilection for affecting male infants [13,33]. RSV hospitalizations are not confined to high-risk groups, as a considerable number also occur among healthy term infants. This finding underscores the widespread impact of RSV across infant subgroups with varying comorbidity profiles. This finding could highlight the need for broader RSV therapeutic strategies, as these healthy infants, though less frequently hospitalized, represent a large segment of the affected population [33]. Simultaneously, a substantial portion of infants who were not otherwise eligible for palivizumab prophylaxis had other comorbidities that could increase the complexity of their care and the potential for adverse outcomes [34].

Our study found that approximately one-quarter of infants were admitted to the ICU during their first RSV-related hospitalization during their first RSV season. In-hospital mortality was rare, occurring in only 0.1%–0.2% of cases. Chest imaging (33.9–38.1%), ECMO use (<0.1–0.2%), supplemental oxygen (14.7–15.7%) administration, and mechanical ventilation (3.7%) were low, despite bronchiolitis guidelines [35]. We emphasize, though, that the accuracy of recorded mechanical ventilation and supplemental oxygen use in insurance claims data might be low, especially for ICU stays shorter than a day. If true, one potential explanation might be that billing for extra procedures necessitates the infant spend a minimum amount of time in the ICU, with billing usually starting on the second day after ICU admission. Conceivably, mechanical ventilation use in an infant requiring it for only a brief period (e.g., less than 24 hours) might not be captured if the infant’s stay in the ICU is brief and the child is transferred to a high-dependency unit or even to the general ward. The contrast between our estimated levels of mechanical ventilation use and those of studies which ascertained ventilation using medical record review also suggests that insurance claims may be inadequate for this purpose. For example, Halasa et al. reviewed medical records to identify mechanical ventilation use in 39 pediatric hospitals across 27 US states and reported that 24% of RSV-hospitalized infants received invasive mechanical ventilation, 25% received non-invasive mechanical ventilation, and 51% received low-flow supplemental oxygen, high-flow nasal cannula oxygen, or continuous positive airway pressure as their highest level of oxygen support [36].

Two other recent studies show evidence of reduced ascertainment of ventilation and/or supplemental oxygen use more broadly in payment claims. Lade et al. found that approximately 6% of infants hospitalized for RSV in Germany required ventilation of any kind (inclusive of oxygen support) [37], while Han et al. estimated that around 2% of infants hospitalized for RSV in South Korea needed mechanical ventilation [38]. Direct quantitative comparisons of this sort may be difficult to interpret given the differing patient mixes, time periods, environments, and operational service definitions reflected across analyses, including ours. Taken together, these results nonetheless seem to support our assertion regarding the limitations of payment claims in this context. Arriola et al., however, reported that 27% of hospitalized children younger than 2 were admitted to the ICU in a subset of FluSurv-NET hospitals, and that 6% received mechanical ventilation [14]. This estimate of mechanical ventilation use was based on clinical records [14,39] and appears to be in line with claims-based estimates. It is worth noting, too, that our estimated proportion of ECMO use may represent a slight underestimate. Recent analyses using the Pediatric Health Information System (PHIS) database [40] observed higher rates of hospitalizations and advanced respiratory support needs post-pandemic, with 70% more children requiring high-flow nasal cannula or noninvasive ventilation, suggesting a broader rise in severe RSV cases requiring intensive care at hospitals offering advanced respiratory services.

The length of inpatient stay for RSV hospitalizations in our study is consistent with the literature, which suggests that the severity of illness and the need for ICU care prolonged hospital stays [41]. Palivizumab-eligible infants had notably longer average lengths of stay, especially when ICU care was required, signifying the heightened complexity of their clinical course and the challenges in managing this high-risk population [42]. Our data also shed light on the variation in healthcare experiences among different comorbidity groups. Palivizumab-eligible infants (comorbidity group B) and infants with other comorbidities (comorbidity group C) faced distinct healthcare challenges, and both experienced elevated risks. Palivizumab-eligible infants had higher utilization of healthcare services, longer lengths of stay, and more days spent in the ICU, highlighting the importance of targeted interventions and close monitoring for high-risk infant populations.

The uniformity in the risk of infants requiring supplemental oxygen, regardless of their comorbidity group, emerges as a salient finding, with a notable emphasis that, while higher-risk infants in Group B are a focus of concern, it is the numerically larger Group A—healthy term infants—that contributes substantially to the overall burden of disease. The discrepancy between the supplemental oxygen use reported in claims datasets (around 15%) and retrospective clinical studies (approximately 70%) indicates a significant under ascertainment of this outcome in our data, suggesting caution when comparing its use across different infant comorbidity profiles [43]. Existing research also emphasizes the significance of effective respiratory support in pediatric patients affected by RSV infections [1]. Furthermore, this consistency may be attributed to the fact that RSV infections primarily manifest as respiratory distress, transcending differences in underlying health conditions [44,45]. While infants with diverse comorbidity profiles may face distinct healthcare challenges and outcomes, the uniformity in supplemental oxygen utilization across claims databases highlights its importance in addressing respiratory distress during RSV episodes. These findings should prompt a deeper exploration of the specific determinants influencing the need for supplemental oxygen across varying clinical contexts. Such investigations could potentially inform tailored approaches to optimize the care and outcomes of RSV-infected infants.

Our study has several limitations. First, the data originate from administrative databases and the use of payment claims data may result in measurement bias and the under ascertainment of some outcomes, such as mechanical ventilation [46–48]. The incidence of oxygen supplementation and mechanical ventilation is relatively infrequent in our study, so it is imperative to consider the possible existence of alternative support (such as heated humidified high-flow therapy) as well as the potential nuances in how these alternatives are reflected in the claims data. Second, the gestational ages of infants, as well as their comorbidity diagnoses, are also subject to misclassification in the claims data we used. Third, it’s plausible that certain infants were incorrectly excluded from our analysis. We identified RSV-related hospitalizations based on the presence of ICD-10-CM diagnosis codes on inpatient medical claims rather than relying on RSV laboratory tests (which are available only for small subsets of infants). This methodology may have led to some misclassification of RSV-related hospitalizations or misattribution of services to an RSV-related hospitalization. We also expect that diagnoses made for the purpose of claims payment might miss RSV-related hospitalizations altogether, so our sample of hospitalizations may not be representative of all RSV-related hospitalizations. We do not feel licensed to speculate, however, about whether our sample would lead us to over- or underestimate inpatient service utilization and length of stay. Finally, though difficult to speculate about their exact nature, differing healthcare and/or coding practices between databases could complicate interpretation of our findings, given observed differences in Census division and payer type between databases. Such inconsistencies could either mask or exaggerate differences in service utilization patterns across the infant populations represented by each database. Our findings also may fail to generalize to populations not covered by the insurance data (e.g., uninsured persons), who might exhibit risk factors or service utilization patterns unlike those captured in commercial and Medicaid claims. Moreover, classifying infants into comorbidity groups may ignore the full spectrum of clinical complexity, and potential variations in coding practices between institutions are not reflected in our results.

Despite these limitations, our findings underscore the need to reduce RSV-related hospitalizations. Future work should attempt to quantify the potential for emerging preventive strategies such as nirsevimab [49] and maternal RSV vaccination [50], both of which aim to protect infants across the comorbidity spectrum, to reduce the severity and cost of RSV hospitalization. Improved access to prevention and healthcare among underserved populations could further enhance these health- and cost-related benefits. Furthermore, the limitations of insurance claims discussed in this section should motivate efforts to improve ascertainment of key outcomes, such as mechanical ventilation, either via targeted data collection or observational data sources (*e*.*g*., electronic medical records) that identify services of interest explicitly.

## Conclusion

In conclusion, our study illustrates healthcare utilization and mortality among infants hospitalized with RSV during their first RSV season. Our findings emphasize the substantial healthcare burden of RSV infections, particularly among high-risk infants. These results have implications for healthcare resource allocation (at the local and systems level), the development of preventive care strategies for RSV, and the need for ongoing research to better understand the epidemiology and clinical course of RSV in infants. Further studies with more comprehensive data sources, explicit identification of healthcare service or procure receipt, and larger sample sizes (particularly for palivizumab-eligible infants) are warranted to refine the understanding of RSV and inform evidence-based interventions.

## Supporting information

S1 Table Codes used to identify MA RSV LRTI index diagnosis using the specific and sensitive definitions. (DOCX)

S2 Table Classification of gestational age groups based on diagnoses attached to birth hospitalization. (DOCX)

S3 Table ICD-10-CM codes assessed for presence of comorbidities. (DOCX)

S4 Table Classification of healthcare service utilization outcomes. (DOCX)

S1 Fig Diagram depicting three example inpatient stays. (DOCX)

S2 Fig Proportion of infants receiving a given service or procedure or dying during RSV related hospitalization, stratified by RSV index diagnosis definition, main versus stability analysis. (DOCX)

S3 Fig Proportion of infants receiving a given service or procedure or dying during RSV-related hospitalization, stratified by RSV index diagnosis definition and comorbidity group, main versus stability analysis. (DOCX)

S4 Fig Length of inpatient stay overall, stratified by RSV index diagnosis definition, main versus stability analysis. (DOCX)

S5 Fig Length of inpatient stay among hospitalizations that did not involve a visit to the ICU, stratified by RSV index diagnosis definition, main versus stability analysis. (DOCX)

S6 Fig Length of inpatient stay among hospitalizations that involved a visit to the ICU, stratified by RSV index diagnosis definition, main versus stability analysis. (DOCX)

S7 Fig Days spent in the ICU among hospitalizations involving a visit to the ICU, stratified by RSV index diagnosis definition, main versus stability analysis. (DOCX)

S8 Fig Length of inpatient stay overall, stratified by RSV index diagnosis definition and comorbidity group, main versus stability analysis. (DOCX)

S9 Fig Length of inpatient stay among hospitalizations that did not involve a visit to the ICU, stratified by RSV index diagnosis definition and comorbidity group, main versus stability analysis. (DOCX)

S10 Fig Length of inpatient stay among hospitalizations that involved a visit to the ICU, stratified by RSV index diagnosis definition and comorbidity group, main versus stability analysis. (DOCX)

S11 Fig Days spent in the ICU among hospitalizations involving a visit to the ICU, stratified by RSV index diagnosis definition and comorbidity group, main versus stability analysis. (DOCX)

S1 File (ZIP)

## Abbreviations

CCAE: Merative MarketScan® Commercial Claims and Encounters
MDCD: MarketScan Multi-State® Medicaid Database
CDM: Optum’s de-identified Clinformatics® Data Mart Database
RSV: respiratory syncytial virus
ICU: intensive care unit
LRTI: lower respiratory tract infection
RSVH: RSV-associated hospitalization
CLD: chronic lung disease
HS-CHD: hemodynamically significant congenital heart disease
ICD-10-CM: International Classification of Diseases, Tenth Revision, Clinical Modification
GA: gestational age
