# Supplementary material for: Inpatient service utilization amongst infants diagnosed with Respiratory Syncytial Virus infection (RSV) in the United States
Source: PLoS One. 2025 Jan 13;20(1):e0317367. doi: 10.1371/journal.pone.0317367 (PMC11730397; doi:10.1371/journal.pone.0317367)
Supplement: S1 Table — (DOCX) [file pone.0317367.s001.docx]

**S2 Table. Codes used to identify MA RSV LRTI index diagnosis using the *specific* and *sensitive* definitions.**

|  |  | **MA RSV LRTI Definition** | |
| --- | --- | --- | --- |
| **ICD-10-CM** | **Description** | **Specific** | **Sensitive** |
| B974*^a^* | Respiratory syncytial virus as the cause of diseases classified elsewhere | x | x |
| J1100 | Influenza with pneumonia, virus not identified |  |  |
| J1108 | Influenza with other manifestations, virus not identified |  |  |
| J111 | Influenza with other respiratory manifestations, virus not identified |  |  |
| J118 | Influenza with other manifestations, virus not identified |  |  |
| J121 | Respiratory syncytial virus pneumonia | x | x |
| J1289 | Viral pneumonia, unspecified |  |  |
| J129 | Viral pneumonia, unspecified |  |  |
| J168 | Pneumonia due to other specified infectious organisms |  |  |
| J17 | Pneumonia diseases classified elsewhere |  |  |
| J180 | Bronchopneumonia, unspecified organism |  |  |
| J181 | Lobar pneumonia, unspecified organism |  |  |
| J182 | Hypostatic pneumonia, unspecified organism |  |  |
| J188 | Other pneumonia, organism unspecified |  |  |
| J189 | Pneumonia, unspecified organism |  |  |
| J205 | Acute bronchitis due to respiratory syncytial virus | x | x |
| J208 | Acute bronchitis due to other specified organisms |  |  |
| J209 | Acute bronchitis, unspecified |  |  |
| J210 | Acute bronchiolitis due to respiratory syncytial virus | x | x |
| J218 | Acute bronchiolitis due to other specified organisms |  | x |
| J219 | Acute bronchiolitis, unspecified |  | x |
| J40 | Bronchitis, not specified as acute or chronic |  |  |
| J22 | Unspecified acute lower respiratory infection |  |  |
| J410 | Simple chronic bronchitis |  |  |
| J411 | Mucopurulent chronic bronchitis |  |  |
| J418 | Mixed simple and mucopurulent chronic bronchitis |  |  |
| J42 | Unspecified chronic bronchitis |  |  |
| J440 | Chronic obstructive pulmonary disease with (acute) lower respiratory infection |  |  |
| J441 | Chronic obstructive pulmonary disease with (acute) exacerbation |  |  |
| J449 | Chronic obstructive pulmonary disease, unspecified |  |  |
| J4520 | Mild intermittent asthma, uncomplicated |  |  |
| J4521 | Mild intermittent asthma with (acute) exacerbation |  |  |
| J4522 | Mild intermittent asthma with status asthmaticus |  |  |
| J4530 | Mild persistent asthma, uncomplicated |  |  |
| J4531 | Mild persistent asthma with (acute) exacerbation |  |  |
| J4532 | Mild persistent asthma with status asthmaticus |  |  |
| J4540 | Moderate persistent asthma, uncomplicated |  |  |
| J4541 | Moderate persistent asthma with (acute) exacerbation |  |  |
| J4542 | Moderate persistent asthma with status asthmaticus |  |  |
| J4550 | Severe persistent asthma, uncomplicated |  |  |
| J4551 | Severe persistent asthma with (acute) exacerbation |  |  |
| J4552 | Severe persistent asthma with status asthmaticus |  |  |
| J4590 | Unspecified asthma |  |  |
| J45901 | Unspecified asthma with (acute) exacerbation |  |  |
| J45902 | Unspecified asthma with status asthmaticus |  |  |
| J45909 | Unspecified asthma, uncomplicated |  |  |
| J4599 | Other asthma |  |  |
| J45990 | Exercise induced bronchospasm |  |  |
| J45991 | Cough variant asthma |  |  |
| J45998 | Other asthma |  |  |
| *Abbreviations:* MA, medically attended; RSV, respiratory syncytial virus; LRTI, lower respiratory tract infection | | | |
| *^a^* In the outpatient and emergency department, B974 was considered a qualifying MA RSV LRTI diagnosis only in conjunction with a Conditional code in the 5 days before or after the date of a diagnosis of B974. All codes in the table above were eligible Conditional codes. Those not indicated as identifying a *Specific* or *Sensitive* MA RSV LRTI diagnosis were used *only* to adjudicate the appearance of B974 in an outpatient or emergency department claim. | | | |

*In order to align analyses across the CCAE, MDCD, and CDM data sets, we considered the first 4 diagnosis positions for diagnoses in the outpatient or emergency room settings and the first 15 diagnosis positions for diagnoses in the inpatient setting.*
