# Supplementary material for: Inpatient service utilization amongst infants diagnosed with Respiratory Syncytial Virus infection (RSV) in the United States
Source: PLoS One. 2025 Jan 13;20(1):e0317367. doi: 10.1371/journal.pone.0317367 (PMC11730397; doi:10.1371/journal.pone.0317367)
Supplement: S2 Table — (DOCX) [file pone.0317367.s002.docx]

**S3 Table. Classification of gestational age groups based on diagnoses attached to birth hospitalization.**

| **Gestational Age** | **Qualifying Codes (Any Position)** |
| --- | --- |
| Preterm, unknown GA | ICD-10-CM P0730: Preterm newborn, unspecified weeks of gestation  DRG 790: Extreme immaturity or respiratory distress syndrome, neonate 791: Prematurity with major problems  792: Prematurity without major problems |
| <29 weeks | ICD-10-CM  P0720: Extreme immaturity of newborn, unspecified weeks of gestation  P0721: Extreme immaturity of newborn, gestational age less than 23 completed weeks  P0722: Extreme immaturity of newborn, gestational age 23 completed weeks  P0723: Extreme immaturity of newborn, gestational age 24 completed weeks  P0724: Extreme immaturity of newborn, gestational age 24 completed weeks  P0725: Extreme immaturity of newborn, gestational age 26 completed weeks  P0726: Extreme immaturity of newborn, gestational age 27 completed weeks  P0731: Preterm newborn, gestational age 28 completed weeks |
| 29–31 weeks | ICD-10-CM  P0732: Preterm newborn, gestational age 29 completed weeks  P0733: Preterm newborn, gestational age 30 completed weeks  P0734: Preterm newborn, gestational age 31 completed weeks |
| 32–36 weeks | ICD-10-CM  P0735: Preterm newborn, gestational age 32 completed weeks  P0736: Preterm newborn, gestational age 33 completed weeks  P0737: Preterm newborn, gestational age 34 completed weeks  P0738: Preterm newborn, gestational age 35 completed weeks  P0739: Preterm newborn, gestational age 36 completed weeks |
| ≥37 weeks | DRG  793: Full term neonate with major problems  795: Normal newborn |
| Unknown | DRG  789: Neonates, died or transferred to another acute care facility  794: Neonate with other significant problems |

*ICD-10-CM, International Classification of Diseases, Tenth Revision, Clinical Modification; DRG, diagnosis related group
Descriptions: ICD-10-CM,* [*https://icd10cmtool.cdc.gov/*](https://icd10cmtool.cdc.gov/)*; DRG,* [*https://www.cms.gov/icd10m/FY2024-nprmversion41.0-fullcode-cms/fullcode_cms/P0380.html*](https://www.cms.gov/icd10m/FY2024-nprmversion41.0-fullcode-cms/fullcode_cms/P0380.html)
