# Supplementary material for: Inpatient service utilization amongst infants diagnosed with Respiratory Syncytial Virus infection (RSV) in the United States
Source: PLoS One. 2025 Jan 13;20(1):e0317367. doi: 10.1371/journal.pone.0317367 (PMC11730397; doi:10.1371/journal.pone.0317367)
Supplement: S3 Table — (DOCX) [file pone.0317367.s003.docx]

**S4 Table. ICD-10-CM codes assessed for presence of comorbidities.**

| **Comorbidity** | **ICD-10-CM codes (Any Position)** |
| --- | --- |
|  |  |
| ***Chronic Lung Disease*** | |
| Chronic respiratory disease arising in the perinatal period | P270; P271; P278; P279 |
| ***Hemodynamically Significant Congenital Heart Disease*** | |
| Higher-risk Congenital Heart Disease | I2783; I420; I425; I428; I429; I5020; I5021; I5022; I5023; I5030; I5031; I5032; I5033; I5040; I5041; I5042; I5043; I50814; I509; Q200; Q201; Q202; Q203; Q204; Q205; Q206; Q208; Q210; Q212; Q213; Q214; Q218; Q220; Q221; Q222; Q224; Q225; Q226; Q228; Q229; Q230; Q231; Q232; Q234; Q240; Q241; Q242; Q243; Q244; Q245; Q248; Q251; Q2521; Q2529; Q253; Q2541; Q2542; Q2543; Q2544; Q2545; Q2546; Q2547; Q2548; Q2549; Q255; Q256; Q2571; Q2572; Q2579; Q258; Q259; Q260; Q261; Q262; Q263; Q264; Q268; Q269 |
| ***Other Comorbid Conditions*** | |
| Congenital and metabolic | E7141; E7142; E7150; E71510; E71511; E71518; E71520; E71521; E71522; E71528; E71529; E7153; E71540; E71541; E71542; E71548; E7400; E7401; E7402; E7403; E7404; E7409; E744; E7521; E7522; E75240; E75241; E75242; E75243; E75248; E75249; E753; E7601; E7602; E7603; E761; E76210; E76211; E76219; E7622; E7629; E763; E768; E769; E770; E771; E778; E779; E7871; E7872; G901; Q000; Q001; Q002; Q010; Q011; Q012; Q018; Q019; Q02; Q030; Q031; Q038; Q039; Q040; Q041; Q042; Q043; Q044; Q045; Q046; Q048; Q049; Q050; Q051; Q052; Q053; Q054; Q055; Q056; Q057; Q058; Q059; Q060; Q061; Q062; Q063; Q064; Q068; Q069; Q0700; Q0701; Q0702; Q0703; Q078; Q079; Q675; Q760; Q761; Q762; Q763; Q76411; Q76412; Q76413; Q76414; Q76415; Q76419; Q76425; Q76426; Q76427; Q76428; Q76429; Q7649; Q790; Q791; Q8711; Q8719; Q872; Q873; Q8740; Q87410; Q87418; Q8742; Q8743; Q875; Q8781; Q8782; Q8789; Q893; Q897; Q898; Q899; Q910; Q911; Q912; Q913; Q914; Q915; Q916; Q917; Q920; Q921; Q922; Q925; Q9261; Q9262; Q927; Q928; Q929; Q930; Q931; Q932; Q933; Q934; Q9351; Q9359; Q937; Q9381; Q9382; Q9388; Q9389; Q939; Q950; Q951; Q952; Q953; Q955; Q958; Q959; Q960; Q961; Q962; Q963; Q964; Q968; Q969; Q970; Q971; Q972; Q973; Q978; Q979; Q980; Q981; Q983; Q984; Q985; Q986; Q987; Q988; Q989; Q990; Q991; Q992; Q998; Q999 |
| Congenital anomalies of the respiratory system | Q300; Q301; Q302; Q303; Q308; Q309; Q310; Q311; Q312; Q313; Q315; Q318; Q319; Q320; Q321; Q322; Q323; Q324; Q330; Q331; Q332; Q333; Q334; Q335; Q336; Q338; Q339; Q340; Q341; Q348; Q349 |
| Cystic fibrosis with pulmonary manifestations | E840; E8411; E8419; E848; E849 |
| Down syndrome without congenital heart disease | Q900; Q901; Q902; Q909 |
| HIV | B9733; B9734; B9735 |
| Immunodeficiency | D800; D801; D802; D803; D804; D805; D806; D807; D808; D809; D810; D811; D812; D8131; D814; D816; D817; D8189; D819; D820; D821; D822; D823; D824; D828; D829; D830; D831; D832; D838; D839; D840; D841; D848; D8481; D84821; D84822; D8489; D849; D893; D8940; D8941; D8942; D8943; D8949; D89810; D89811; D89812; D89813; D8982; D89831; D89832; D89833; D89834; D89835; D89839; D8989; D899; M359 |
| Lower-risk congenital heart disease | P2930; P2938; Q209; Q211; Q219; Q223; Q233; Q238; Q239; Q246; Q249; Q250; Q2540 |
| Neuromuscular disease | E7500; E7501; E7502; E7509; E7510; E7511; E7519; E7523; E7525; E7526; E7529; E754; F842; G120; G121; G1220; G1221; G1222; G1223; G1224; G1225; G1229; G128; G129; G3181; G3182; G319; G600; G601; G602; G603; G608; G609; G7100; G7101; G7102; G7109; G7111; G7112; G7113; G712; G7120; G7121; G71220; G71228; G7129; G719; G729; G733; G800; G801; G802; G804; G808; G809; G9389; G939; P940 |
| *ICD-10-CM, International Classification of Diseases, Tenth Revision, Clinical Modification*  *A case was considered to have a given comorbidity if evidence of a diagnosis occurred prior to the date of their MA RSV LRTI diagnosis (assessed separately for the sensitive and specific LRTI diagnoses).* | |
