# Supplementary material for: Inpatient service utilization amongst infants diagnosed with Respiratory Syncytial Virus infection (RSV) in the United States
Source: PLoS One. 2025 Jan 13;20(1):e0317367. doi: 10.1371/journal.pone.0317367 (PMC11730397; doi:10.1371/journal.pone.0317367)
Supplement: S4 Table — (DOCX) [file pone.0317367.s004.docx]

**S5 Table.** **Classification of healthcare service utilization outcomes.**

| **Outcome** | **Qualifying Codes** | **Condition / *Notes*** |
| --- | --- | --- |
| Intensive Care Unit | **Revenue Center Code** 0200: General classification for ICU  0201: Surgical ICU  0202: Medical ICU  0203: Pediatric ICU  0204: Psychiatric ICU  0208: Trauma ICU  0209: Other ICU | At least one code found between inpatient admission date and discharge date (inclusive) |
| Supplemental Oxygen Use,  In-Hospital | **CPT**  94660: Continuous positive airway pressure ventilation (CPAP), initiation and management  94640: Treatment of acute airway obstruction with inhaled medication and/or the use of an inhalation treatment to induce sputum for diagnostic purposes.  **HCPCS**  E1399: Durable medical equipment, miscellaneous  E1405: Oxygen and water vapor enriching system with heated delivery  E1406: Oxygen and water vapor enriching system without heated delivery  A4608: Transtracheal oxygen catheter  A4606*: Oxygen probe for use with oximeter device, replacement  A4619*: Face tent  A4620*: Variable concentration mask  A4615*: Cannula, nasal  A4616*: Tubing (oxygen), per foot  A4617*: Mouth piece | At least one code found between inpatient admission date and discharge date (inclusive)  ** Codes also appear in discharge to home with supplemental oxygen.* |
| Supplemental Oxygen Use,  Post Discharge | **HCPCS** E0424: Stationary compressed gaseous oxygen system, rental; includes container, contents, regulator, flowmeter, humidifier, nebulizer, cannula or mask, and tubing  E0425: Stationary compressed gas system, purchase; includes regulator, flowmeter, humidifier, nebulizer, cannula or mask, and tubing  E0430: Portable gaseous oxygen system, purchase; includes regulator, flowmeter, humidifier, cannula or mask, and tubing  E0431: Portable gaseous oxygen system, rental; includes portable container, regulator, flowmeter, humidifier, cannula or mask, and tubing  E0433: Portable liquid oxygen system, rental; home liquefier used to fill portable liquid oxygen containers, includes portable containers, regulator, flowmeter, humidifier, cannula or mask and tubing, with or without supply reservoir and contents gauge  E0434: Portable liquid oxygen system, rental; includes portable container, supply reservoir, humidifier, flowmeter, refill adaptor, contents gauge, cannula or mask, and tubing  E0435: Portable liquid oxygen system, purchase; includes portable container, supply reservoir, flowmeter, humidifier, contents gauge, cannula or mask, tubing and refill adaptor  E0439: Stationary liquid oxygen system, rental; includes container, contents, regulator, flowmeter, humidifier, nebulizer, cannula or mask, & tubing  E0440: Stationary liquid oxygen system, purchase; includes use of reservoir, contents indicator, regulator, flowmeter, humidifier, nebulizer, cannula or mask, and tubing  E0441: Stationary oxygen contents, gaseous, 1 month's supply = 1 unit  E0442: Stationary oxygen contents, liquid, 1 month's supply = 1 unit  E0443: Portable oxygen contents, gaseous, 1 month's supply = 1 unit  E0444: Portable oxygen contents, liquid, 1 month's supply = 1 unit  E0445: Oximeter device for measuring blood oxygen levels non-invasively  E0446: Topical oxygen delivery system, not otherwise specified, includes all supplies and accessories  E0447: Portable oxygen contents, liquid, 1 month's supply = 1 unit, prescribed amount at rest or nighttime exceeds 4 liters per minute (lpm)  E0465: Home ventilator, any type, used with invasive interface, (e.g., tracheostomy tube)  E0466: Home ventilator, any type, used with non-invasive interface, (e.g., mask, chest shell)  E0467: Home ventilator, multi-function respiratory device, also performs any or all of the additional functions of oxygen concentration, drug nebulization, aspiration, and cough stimulation, includes all accessories, components and supplies for all functions  E0470: Respiratory assist device, bi-level pressure capability, without backup rate feature, used with noninvasive interface, e.g., nasal or facial mask (intermittent assist device with continuous positive airway pressure device)  E0471: Respiratory assist device, bi-level pressure capability, with back-up rate feature, used with noninvasive interface, e.g., nasal or facial mask (intermittent assist device with continuous positive airway pressure device)  E0472: Respiratory assist device, bi-level pressure capability, with backup rate feature, used with invasive interface, e.g., tracheostomy tube (intermittent assist device with continuous positive airway pressure device)  E1390: Oxygen concentrator, single delivery port, capable of delivering 85 percent or greater oxygen concentration at the prescribed flow rate  E1391: Oxygen concentrator, dual delivery port, capable of delivering 85 percent or greater oxygen concentration at the prescribed flow rate, each  E1392: Portable oxygen concentrator, rental  E1352: Oxygen accessory, flow regulator capable of positive inspiratory pressure  E1353: Regulator  E1354: Oxygen accessory, wheeled cart for portable cylinder or portable concentrator, any type, replacement only, each  E1355: Stand/rack  E1356: Oxygen accessory, battery pack/cartridge for portable concentrator, any type, replacement only, each  E1357: Oxygen accessory, battery charger for portable concentrator, any type, replacement only, each  E1358: Oxygen accessory, dc power adapter for portable concentrator, any type, replacement only, each  E1372: Immersion external heater for nebulizer  0277: Medical/Surgical Supplies and Devices (also see 062X, an extension of 027X) Oxygen-Take Home  0413: Respiratory Services Hyperbaric Oxygen Therapy  0544: Ambulance Oxygen  0600: Home Health (HH) - Oxygen General Classification  0601: Home Health (HH) - Oxygen Oxygen - Stat Equip/Supply/Content  0602: Home Health (HH) - Oxygen Oxygen - Stat Equip/Supply<1 LPM  0603: Home Health (HH) - Oxygen Oxygen - Stat Equip/Supply>4 LPM  0604: Home Health (HH) - Oxygen Oxygen - Port Add-on  0609: Home Health (HH) - Oxygen Oxygen - Other  A4611: Battery, heavy-duty; replacement for patient-owned ventilator  A4612: Battery cables; replacement for patient-owned ventilator  A4613: Battery charger; replacement for patient-owned ventilator  E0465: Home ventilator, any type, used with invasive interface, (e.g., tracheostomy tube)  E0466: Home ventilator, any type, used with non-invasive interface, (e.g., mask, chest shell)  E0467: Home ventilator, multi-function respiratory device, also performs any or all of the additional functions of oxygen concentration, drug nebulization, aspiration, and cough stimulation, includes all accessories, components and supplies for all functions  A4483: Moisture exchanger, disposable, for use with invasive mechanical ventilation  A4606*: Oxygen probe for use with oximeter device, replacement  A4619*: Face tent  A4620*: Variable concentration mask  A4615*: Cannula, nasal  A4616*: Tubing (oxygen), per foot  A4617*: Mouth piece | At least one code found between inpatient discharge date and discharge date + 7 days (inclusive)  ** Codes also appear in in-hospital supplemental oxygen.* |
| Mechanical Ventilation,  Non-Invasive | **ICD-10-PCS**  5A0935*x*: Assistance with Respiratory Ventilation, Less than 24 Consecutive Hours, Continuous Positive Airway Pressure  5A0945*x*: Assistance with Respiratory Ventilation, 24-96 Consecutive Hours, Continuous Positive Airway Pressure  5A0955*x*: Assistance with Respiratory Ventilation, Greater than 96 Consecutive Hours, Continuous Positive Airway Pressure  **CPT**  94660: Continuous positive airway pressure ventilation (CPAP), initiation and management  94662: CPAP ventilation management  94002: Ventilation assist and management, initiation of pressure or volume preset ventilators for assisted or controlled breathing, hospital inpatient/observation, initial day  94003: Hospital inpatient/observation, each subsequent day  **HCPCS**  E0601: Continuous positive airway pressure (CPAP) device  A4618: Breathing circuits | At least one code found between inpatient admission date and discharge date (inclusive)  Code qualified if 0B21XEZ, 0BH17EZ, 0BH18EZ, or 0BH13EZ not found ≤ 1 day prior to service date OR within the start/end dates of a given service. |
| Mechanical Ventilation,  Invasive | ICD-10-PCS  0B21XEZ: Change Endotracheal Airway in Trachea, External Approach  0BH17EZ: Insertion of Endotracheal Airway into Trachea, Via Opening  0BH18EZ: Insertion of Endotracheal Airway into Trachea, Endo  0BH13EZ: Insertion of Endotracheal Airway into Trachea, Perc Approach  5A1935Z: Respiratory Ventilation, Less than 24 Consecutive Hours  5A1945Z: Respiratory Ventilation, 24-96 Consecutive Hours  5A1955Z: Respiratory Ventilation, Greater than 96 Consecutive Hours  5A0935*x*: Assistance with Respiratory Ventilation, Less than 24 Consecutive Hours, Continuous Positive Airway Pressure  5A0945*x*: Assistance with Respiratory Ventilation, 24-96 Consecutive Hours, Continuous Positive Airway Pressure  5A0955*x*: Assistance with Respiratory Ventilation, Greater than 96 Consecutive Hours, Continuous Positive Airway Pressure  **CPT**  94660: Continuous positive airway pressure ventilation (CPAP), initiation and management  94662: CPAP ventilation management  94002: Ventilation assist and management, initiation of pressure or volume preset ventilators for assisted or controlled breathing, hospital inpatient/observation, initial day  94003: Hospital inpatient/observation, each subsequent day  **HCPCS**  E0601: Continuous positive airway pressure (CPAP) device  A4618: Breathing circuits | At least one code found between inpatient admission date and discharge date (inclusive)  *Grayed Out:* Code qualified if 0B21XEZ, 0BH17EZ, 0BH18EZ, or 0BH13EZ found ≤ 1 day prior to service date.  *x*, includes child codes |
| Extracorporeal Membrane Oxygenation | **ICD-10-PCS**  5A1522*x*: Extracorporeal Oxygenation, Membrane  **CPT**  36822: Insertion of cannula(s) for prolonged extracorporeal circulation for cardiopulmonary insufficiency (ECMO) (separate procedure)  33960: Prolonged extracorporeal circulation for cardiopulmonary insufficiency; initial day  33961: Prolonged extracorporeal circulation for cardiopulmonary insufficiency; each subsequent day  33946: Extracorporeal membrane oxygenation (ECMO)/extracorporeal life support (ECLS) provided by physician; initiation, veno-venous  33947: Extracorporeal membrane oxygenation (ECMO)/extracorporeal life support (ECLS) provided by physician; initiation, veno-arterial.  33948: Extracorporeal membrane oxygenation (ECMO)/extracorporeal life support (ECLS) provided by physician; daily management, each day, veno-venous  33949: Extracorporeal membrane oxygenation (ECMO)/extracorporeal life support (ECLS) provided by physician; daily management, each day, veno-arterial.  33951: Extracorporeal membrane oxygenation (ECMO)/extracorporeal life support (ECLS) provided by physician; insertion of peripheral (arterial and/or venous) cannula(e), percutaneous, birth through 5 years of age (includes fluoroscopic guidance, when performed  33953: Extracorporeal membrane oxygenation (ECMO)/extracorporeal life support (ECLS) provided by physician; insertion of peripheral (arterial and/or venous) cannula(e), open, birth through 5 years of age  33955: Extracorporeal membrane oxygenation (ECMO)/extracorporeal life support (ECLS) provided by physician; insertion of central cannula(e) by sternotomy or thoracotomy, birth through 5 years of age  33957: Extracorporeal membrane oxygenation (ECMO)/extracorporeal life support (ECLS) provided by physician; reposition peripheral (arterial and/or venous) cannula(e), percutaneous, birth through 5 years of age (includes fluoroscopic guidance, when performed)  33959: Extracorporeal membrane oxygenation (ECMO)/extracorporeal life support (ECLS) provided by physician; reposition central (arterial and/or venous) cannula(e), open, birth through 5 years of age. (includes fluoroscopic guidance, when performed).  33963: Extracorporeal membrane oxygenation (ECMO)/extracorporeal life support (ECLS) provided by physician; reposition central cannula(e) by sternotomy or thoracotomy, birth through 5 years of age. (includes fluoroscopic guidance, when performed).  33965: Extracorporeal membrane oxygenation (ECMO)/extracorporeal life support (ECLS) provided by physician; removal of peripheral (arterial and/or venous) cannula(e), percutaneous, birth through 5 years of age.  33969: Extracorporeal membrane oxygenation (ECMO)/extracorporeal life support (ECLS) provided by physician; removal of peripheral (arterial and/or venous) cannula(e), open, birth through 5 years of age.  33985: Extracorporeal membrane oxygenation (ECMO)/extracorporeal life support (ECLS) provided by physician; removal of central cannula(e) by sternotomy or thoracotomy, birth through 5 years of age. | At least one code found between inpatient admission date and discharge date (inclusive)  *x*, includes child codes |
| Chest Imaging | **CPT** 71045: Radiologic examination, chest; single view  71046: Radiologic examination, chest; 2 views  71047: Radiologic examination, chest; 3 views  71048: Radiologic examination, chest; 4 or more views  71250: CT Chest without contrast material  71260: CT Chest with contrast material  71270: CT Chest with and without contrast material  71550: Chest w/ and w/o contrast  71551: Chest w/ contrast  71552: Chest Wall/Rib, Sternum, Bilateral Pectoralis Muscles, Bilateral Clavicles w/ and w/o contrast  715555C: MRA/MRV Chest w/ and w/o contrast  715555A: MRA/MRV Chest w/o contrast  78811: PET imaging, limited area  78812: PET imaging, skull to mid-thigh  78813: PET imaging, whole body | At least one code found between inpatient admission date and discharge date (inclusive) |

*ICU, Intensive Care Unit*

*CPT, Current Procedural Terminology ®*

*HCPCS, Healthcare Common Procedure Coding System*

*ICD-10-PCS, ICD-10 Procedure Coding System*

*ECMO, extracorporeal membrane oxygenation*
