# Supplementary material for: Inpatient service utilization amongst infants diagnosed with Respiratory Syncytial Virus infection (RSV) in the United States
Source: PLoS One. 2025 Jan 13;20(1):e0317367. doi: 10.1371/journal.pone.0317367 (PMC11730397; doi:10.1371/journal.pone.0317367)
Supplement: S1 Fig — (DOCX) [file pone.0317367.s005.docx]

**S1 Figure. Diagram depicting three example inpatient stays.**

**
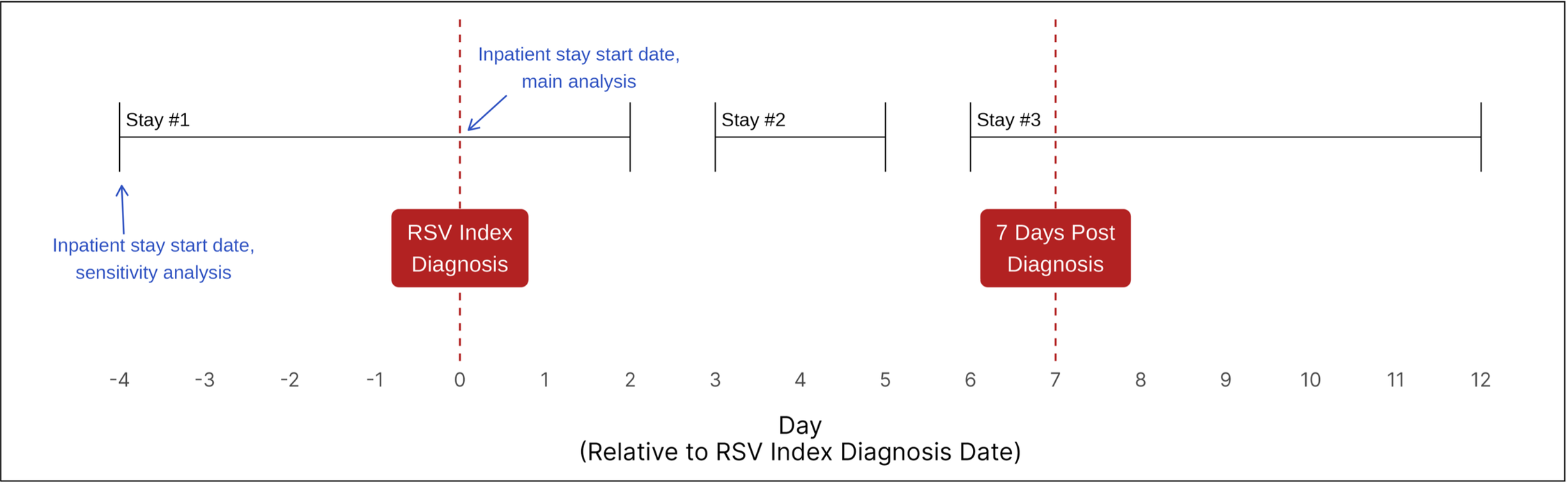
**

*All three of the inpatient stays labeled in the diagram would have been included as RSV-related hospitalizations in our analysis, based on their proximity to the RSV index diagnosis definition. The x-axis depicts the day relative to the date of the RSV index diagnosis (day 0). Blue labels indicate the alternative start dates for Stay #1 in the main and sensitivity analyses, given that the RSV index diagnosis occurs more than 3 days after inpatient admission.*
