# Supplementary material for: Inpatient service utilization amongst infants diagnosed with Respiratory Syncytial Virus infection (RSV) in the United States
Source: PLoS One. 2025 Jan 13;20(1):e0317367. doi: 10.1371/journal.pone.0317367 (PMC11730397; doi:10.1371/journal.pone.0317367)
Supplement: S2 Fig — (DOCX) [file pone.0317367.s006.docx]

**S6 Figure. Proportion of infants receiving a given service or procedure or dying during RSV-related hospitalization, stratified by RSV index diagnosis definition, main versus stability analysis.**


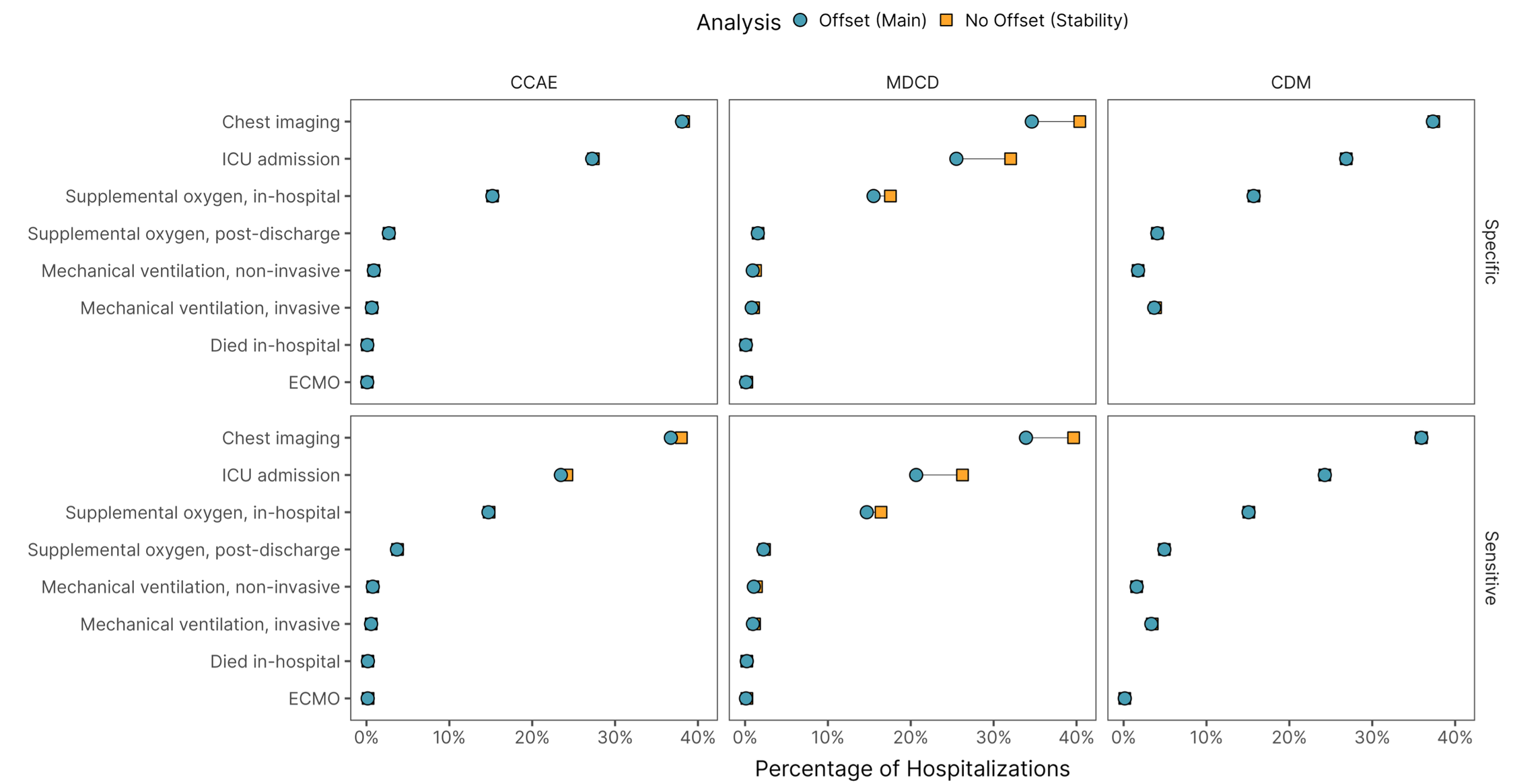


*In the main analysis, we left-truncated the inpatient when the RSV index diagnosis occurred more than three days into an inpatient stay, while in the stability analysis, we retained the original start date of the inpatient stay. Select outcomes are suppressed in the CDM panels due to potentially derivable small cell sizes. CCAE, MarketScan Commercial; MDCD, Multi-State Medicaid; CDM, Clinformatics®.*
