# Supplementary material for: Inpatient service utilization amongst infants diagnosed with Respiratory Syncytial Virus infection (RSV) in the United States
Source: PLoS One. 2025 Jan 13;20(1):e0317367. doi: 10.1371/journal.pone.0317367 (PMC11730397; doi:10.1371/journal.pone.0317367)
Supplement: S4 Fig — (DOCX) [file pone.0317367.s008.docx]

**S8 Figure. Length of inpatient stay overall, stratified by RSV index diagnosis definition, main versus stability analysis.**


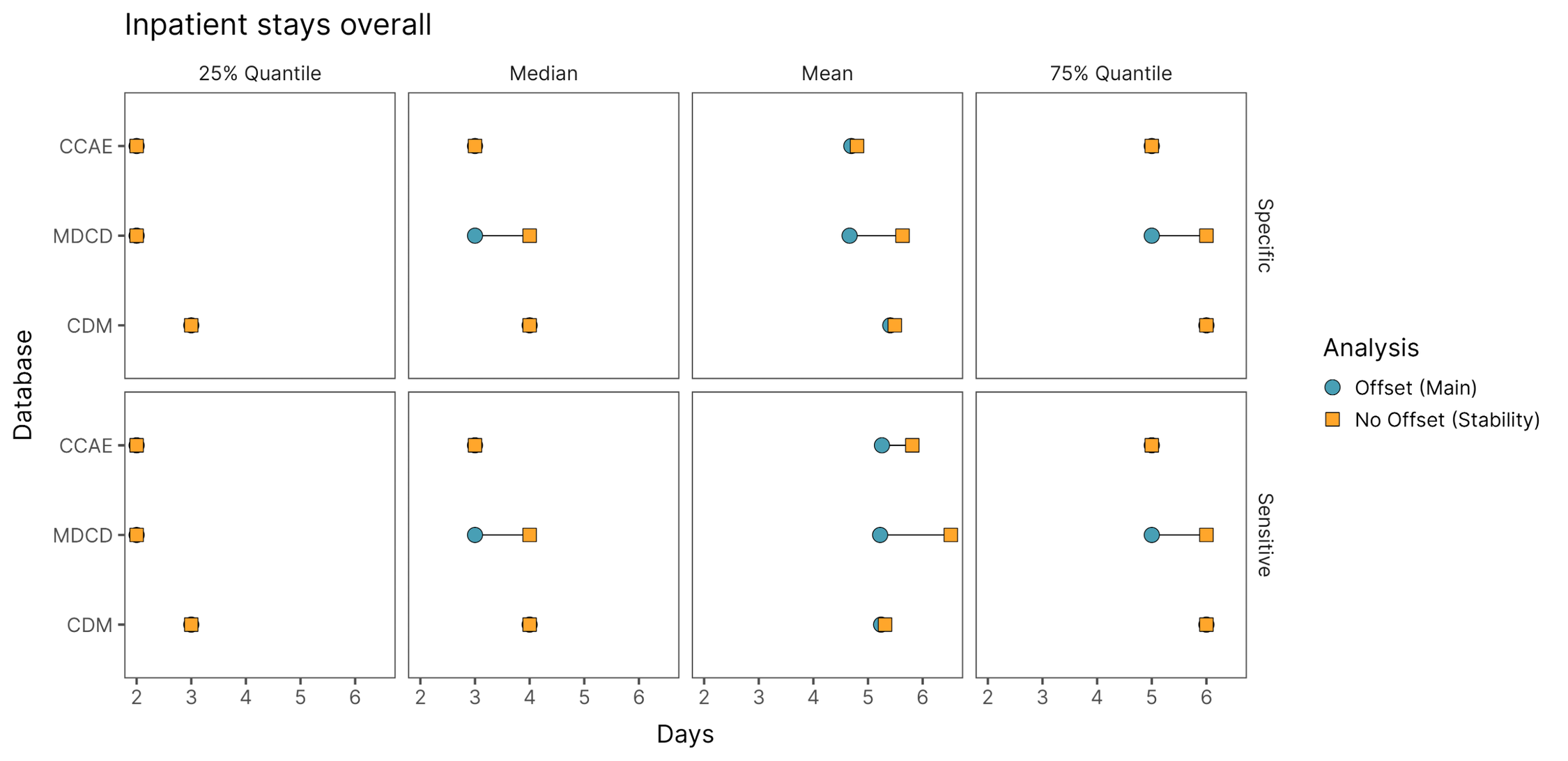


*In the main analysis, we left-truncated the inpatient when the RSV index diagnosis occurred more than three days into an inpatient stay, while in the stability analysis, we retained the original start date of the inpatient stay. CCAE, MarketScan Commercial; MDCD, Multi-State Medicaid; CDM, Clinformatics®.*
