# Supplementary material for: Inpatient service utilization amongst infants diagnosed with Respiratory Syncytial Virus infection (RSV) in the United States
Source: PLoS One. 2025 Jan 13;20(1):e0317367. doi: 10.1371/journal.pone.0317367 (PMC11730397; doi:10.1371/journal.pone.0317367)
Supplement: S9 Fig — (DOCX) [file pone.0317367.s013.docx]

**S13 Figure. Length of inpatient stay among hospitalizations that did not involve a visit to the ICU, stratified by RSV index diagnosis definition and comorbidity group, main versus stability analysis.**


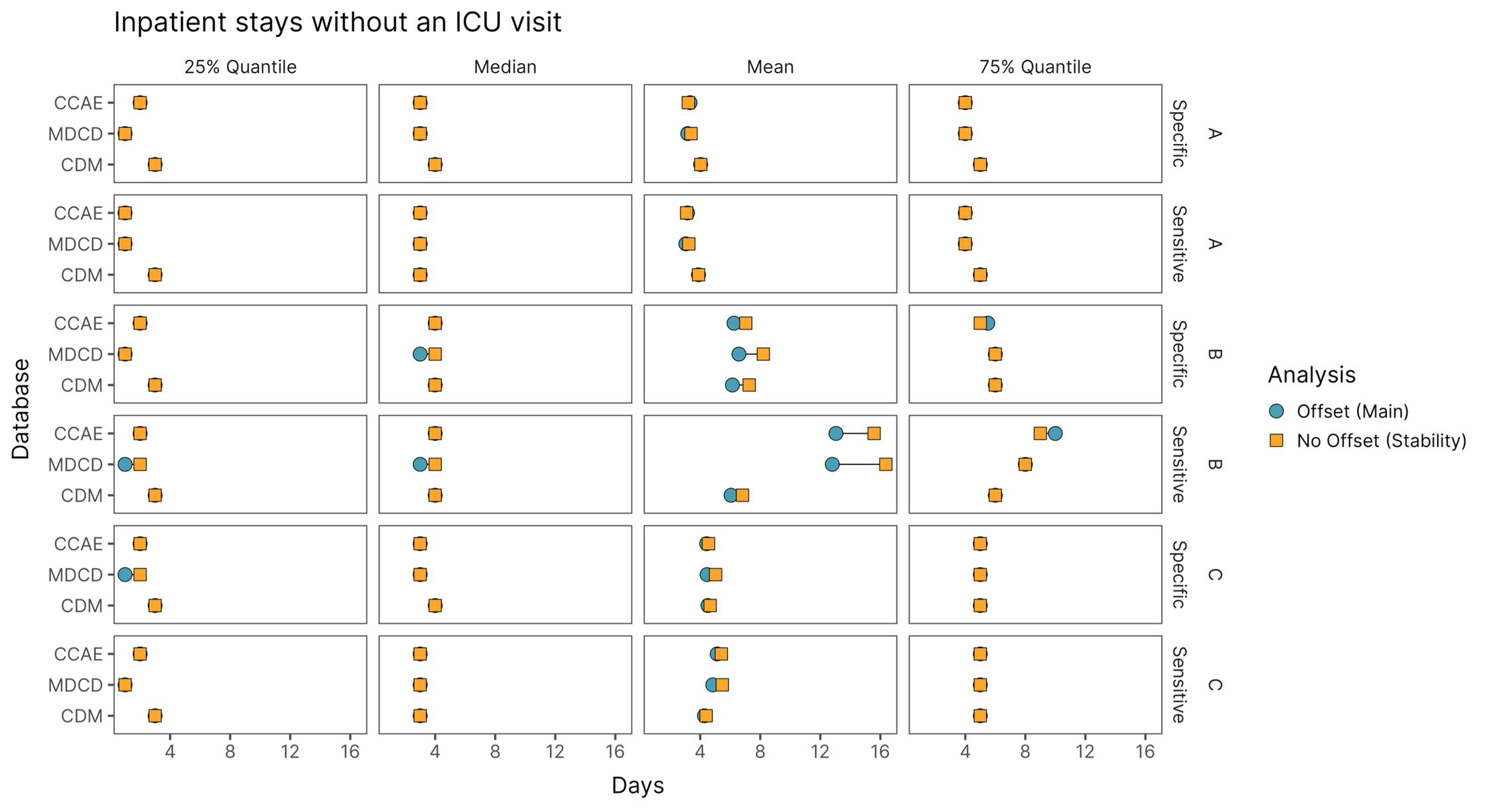


*In the main analysis, we left-truncated the inpatient when the RSV index diagnosis occurred more than three days into an inpatient stay, while in the stability analysis, we retained the original start date of the inpatient stay. CCAE, MarketScan Commercial; MDCD, Multi-State Medicaid; CDM, Clinformatics®.*
