# Supplementary material for: Inpatient service utilization amongst infants diagnosed with Respiratory Syncytial Virus infection (RSV) in the United States
Source: PLoS One. 2025 Jan 13;20(1):e0317367. doi: 10.1371/journal.pone.0317367 (PMC11730397; doi:10.1371/journal.pone.0317367)
Supplement: S11 Fig — (DOCX) [file pone.0317367.s015.docx]

**S13 Figure. Length of inpatient stay among hospitalizations that did not involve a visit to the ICU, stratified by RSV index diagnosis definition and comorbidity group, main versus stability analysis.**


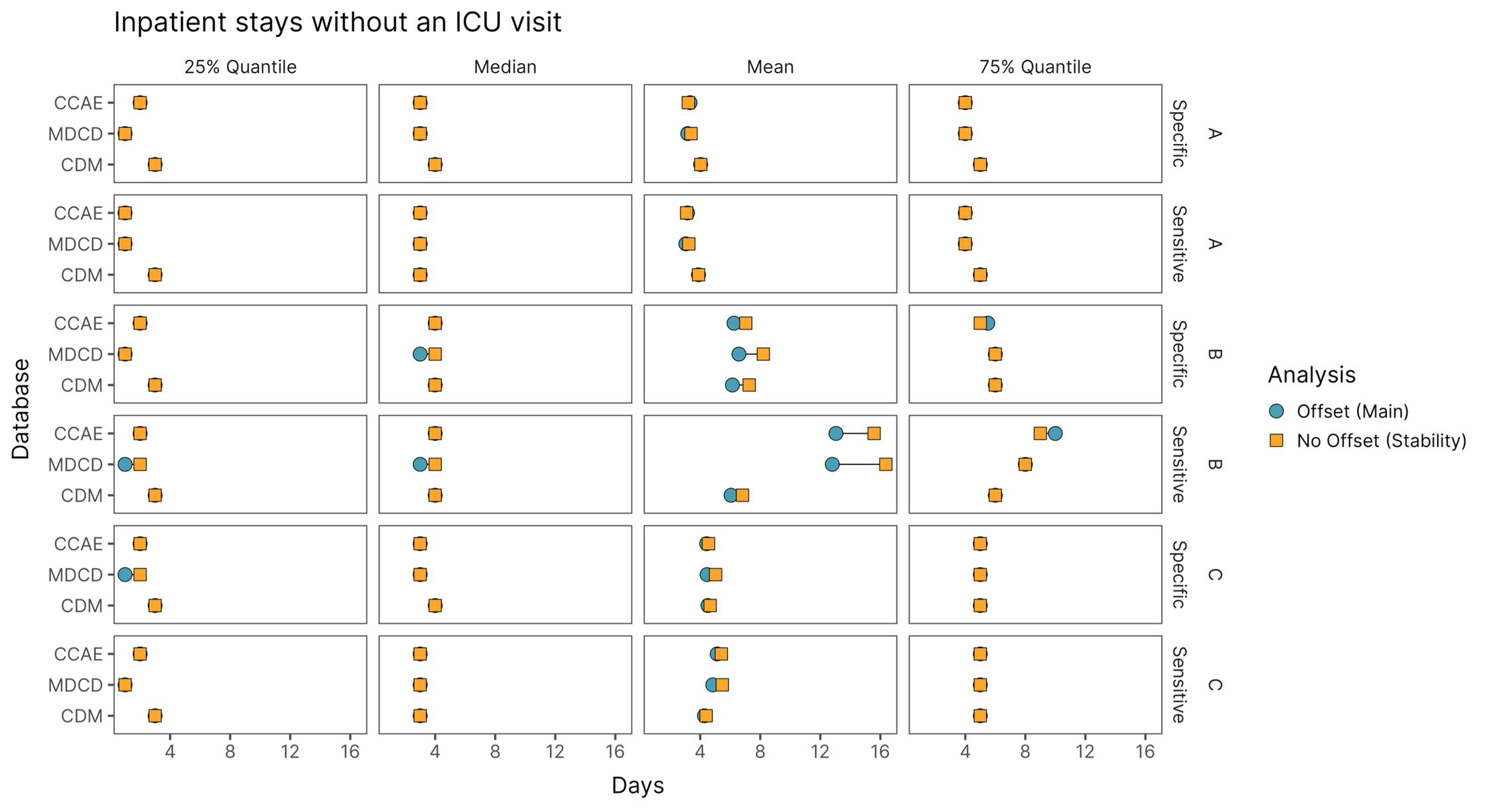


*In the main analysis, we left-truncated the inpatient when the RSV index diagnosis occurred more than three days into an inpatient stay, while in the stability analysis, we retained the original start date of the inpatient stay. CCAE, MarketScan Commercial; MDCD, Multi-State Medicaid; CDM, Clinformatics®.*

**S15 Figure. Days spent in the ICU among hospitalizations involving a visit to the ICU, stratified by RSV index diagnosis definition and comorbidity group, main versus stability analysis.**

NOTE: Numeric values from Clinformatics (CDM) shown in this figure come from a different output export than the rest of the CDM data shown throughout the paper and supplement. Code corrections used to produce these values may not have been saved in the final repository, though only the CDM values shown in this figure would have been affected. Please see the section entitled “Corrected S15 Figure Numbers (CDM)” in the README.txt file within S16 Code for details.


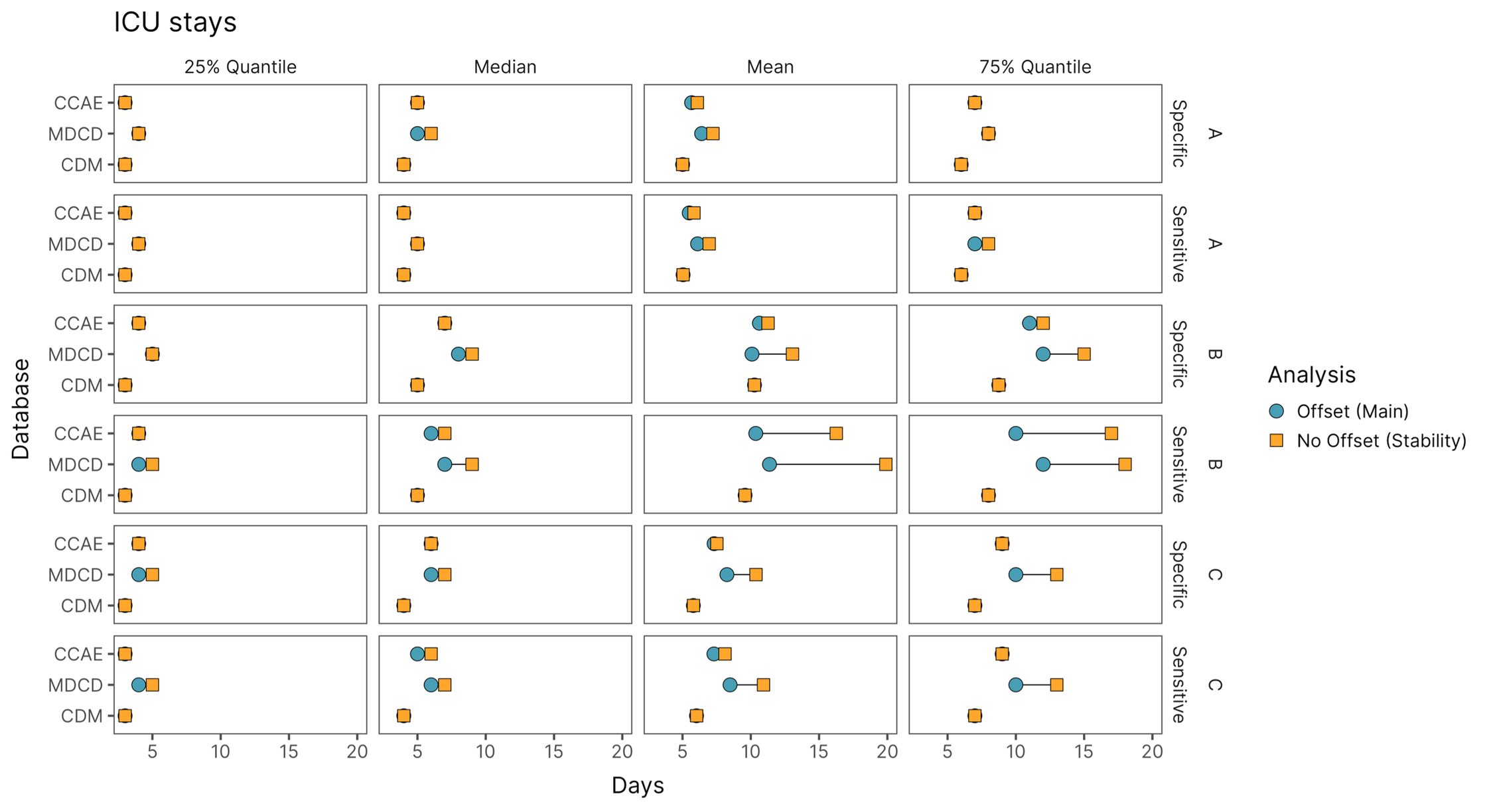


*In the main analysis, we left-truncated the inpatient when the RSV index diagnosis occurred more than three days into an inpatient stay, while in the stability analysis, we retained the original start date of the inpatient stay. CCAE, MarketScan Commercial; MDCD, Multi-State Medicaid; CDM, Clinformatics®.*
